# Widespread infection, diversification and old host associations of *Nosema* Microsporidia in European freshwater gammarids (Amphipoda)

**DOI:** 10.1371/journal.ppat.1011560

**Published:** 2023-08-21

**Authors:** Karolina Bacela-Spychalska, Remi Wattier, Maria Teixeira, Richard Cordaux, Adrien Quiles, Michal Grabowski, Piotr Wroblewski, Mykola Ovcharenko, Daniel Grabner, Dieter Weber, Alexander M. Weigand, Thierry Rigaud

**Affiliations:** 1 Department of Invertebrate Zoology and Hydrobiology, Faculty of Biology and Environmental Protection, University of Lodz, Lodz, Poland; 2 Laboratoire Biogéosciences, UMR CNRS 6282, Université de Bourgogne, Dijon, France; 3 Laboratoire Ecologie et Biologie des Interactions, Equipe Ecologie Evolution Symbiose, UMR CNRS 7267, Université de Poitiers, Poitiers, France; 4 Department of Ecology and Evolution of Parasitism, Witold Stefanski Institute of Parasitology, Polish Academy of Science, Warsaw, Poland; 5 Institute of Biology and Earth Sciences, Pomeranian University in Slupsk, Slupsk, Poland; 6 Aquatic Ecology, University of Duisburg-Essen, Essen, Germany; 7 Centre for Water and Environmental Research, University of Duisburg-Essen, Essen, Germany; 8 Senckenberg Deutsches Entomologisches Institut Müncheberg, Germany; 9 Musée National d’Histoire Naturelle Luxembourg, Luxembourg, Luxembourg; University of California Riverside, UNITED STATES

## Abstract

The microsporidian genus *Nosema* is primarily known to infect insects of economic importance stimulating high research interest, while other hosts remain understudied. *Nosema granulosis* is one of the formally described *Nosema* species infecting amphipod crustaceans, being known to infect only two host species. Our first aim was to characterize *Nosema* spp. infections in different amphipod species from various European localities using the small subunit ribosomal DNA (SSU) marker. Second, we aimed to assess the phylogenetic diversity, host specificity and to explore the evolutionary history that may explain the diversity of gammarid-infecting *Nosema* lineages by performing a phylogenetic reconstruction based on RNA polymerase II subunit B1 (RPB1) gene sequences. For the host species *Gammarus balcanicus*, we also analyzed whether parasites were in excess in females to test for sex ratio distortion in relation with *Nosema* infection. We identified *Nosema* spp. in 316 individuals from nine amphipod species being widespread in Europe. The RPB1-based phylogenetic reconstruction using newly reported sequences and available data from other invertebrates identified 39 haplogroups being associated with amphipods. These haplogroups clustered into five clades (A-E) that did not form a single amphipod-infecting monophyletic group. Closely related sister clades C and D correspond to *Nosema granulosis*. Clades A, B and E might represent unknown *Nosema* species infecting amphipods. Host specificity seemed to be variable with some clades being restricted to single hosts, and some that could be found in several host species. We show that *Nosema* parasite richness in gammarid hosts is much higher than expected, illustrating the advantage of the use of RPB1 marker over SSU. Finally, we found no hint of sex ratio distortion in *Nosema* clade A infecting *G*. *balcanicus*. This study shows that *Nosema* spp. are abundant, widespread and diverse in European gammarids. Thus, *Nosema* is as diverse in aquatic as in terrestrial hosts.

## Author summary

The intracellular microsporidian parasites of the genus *Nosema* are important pathogens as they infect insects of economic importance such as pollinators or domestic silk moths. However, they have been overlooked in other groups of invertebrates such as crustaceans, where only few *Nosema* spp. were detected, including *Nosema granulosis* in two gammarid amphipod hosts. Here, using molecular tools, we detected infections by *Nosema* spp. in nine freshwater amphipod species from various European localities. These infections are common, widespread, and the phylogenetic analysis revealed that some *Nosema* lineages are specific to certain host species, while the others are not restricted to single hosts. We also confirms that there are several *Nosema* lineages infecting gammarid crustaceans, not only *N*. *granulosis*, that can be new candidate species of *Nosema*. We conclude that *Nosema* is as diverse in aquatic as in terrestrial hosts.

## Introduction

Since the mid-19th century, Microsporidia, a large group of obligate intracellular eukaryotic microparasites related to fungi, has aroused interest in both basic and applied studies [1–3]. In this context, the microsporidian genus *Nosema* (Nosematida) holds in a seminal place. The first formally described Microsporidium was *Nosema bombycis* [4]. Since then, 16 *Nosema* spp. or closely-related species have been described infecting insects [5]. All are pathogenic to some extent, with some species having a negative economic impact and some have been used for insect pest control [6,7]. There are strains of *Nosema* that are horizontally transmitted (HT) [8], while others are known to also use vertical transmission (VT) in their life cycle [9].

Few *Nosema* spp. are known to infect freshwater decapod or amphipod crustaceans based on morphological and molecular data [10–18]. Many amphipod species are known to serve as hosts for many other microsporidian parasites, such as *Dictyocoela* spp. and *Cucumispora* spp. [summarised in 18], and one *Nosema* species, *N*. *granulosis*, which has been reported from only two amphipod species. This parasite, unlike *Nosema* of insects, has not been recognized as a pathogen so far, while in other crustaceans the impact on host fitness is unknown. *Nosema granulosis* was first described in the amphipod *Gammarus duebeni* [10]. Infected *G*. *duebeni* females transmit *N*. *granulosis* directly to their offspring through infected eggs [19–21]. Vertical transmission occurs only via female hosts because eggs contain enough cytoplasm in which *N*. *granulosis* can live and be transmitted [22]. Under the selective pressure of this transmission asymmetry, traits increasing the proportion of female hosts is selectively advantageous for the parasites. Therefore, the parasite’s ability for turning genetic males into functional females (feminization) was selected [10,23–26]. As a consequence of this feminization, female-biased sex ratios can be found in populations of *G*. *duebeni* and *Gammarus roeselii* [20,21,23,27] and only a few males were found to be infected by *N*. *granulosis* in *G*. *roeselii* (presumably resulting from failed feminisation) [23]. These vertically-transmitted infections cause little pathogenicity to their hosts [10,23,28,29], even having beneficial effects in *G*. *roeselii* [23]. Because of female excess and positive effect on fitness-related traits, host populations infected with the feminizing *N*. *granulosis* strains are predicted to have higher growth rate and may help the invasive host *G*. *roeselii* in colonizing new territories [30]. Supporting this hypothesis, Quiles *et al*. [31] found that the feminizing *N*. *granulosis* strain of *G*. *roeselii* is associated with the only one host mitochondrial genotype that invaded Western Europe after the last glaciation [32]. However, as feminizing *Dictyocoela* microsporidians vary in their feminizing ability [21], it is likewise possible that the feminization capacity of *N*. *granulosis* infecting *G*. *duebeni* and *G*. *roeselii* varies between parasite strains and/or host species. The recent detection of *Nosema* spp. in populations of different amphipod species with highly variable prevalence fuels this hypothesis [33,34], but more data are necessary to understand *N*. *granulosis* diversity in amphipods. Studying microsporidian infections in amphipods is facilitated by the fact that some hosts are subjected to intensive phylogenetic and biogeographical research. For example, a study on New Zealand amphipods suggested that amphipod-*Dictyocoela* associations could have preceded the split of Pangea some 180 million years ago (MYA) and that these parasites co-differentiated with their hosts [35]. Similarly, the resolution of the evolutionary histories of morphospecies *Gammarus balcanicus* and *G*. *roeselii* [32,36–38] allowed the study of co-differentiation between these hosts and some of their microsporidian parasites, especially those belonging to the genus *Dictyocoela* [31,39,40]. However, data on *Nosema* infections remain too scarce in European gammarids to allow a general analysis of their diversification. *Gammarus balcanicus* is a potential host of particular interest, having begun its diversification in the early Miocene (c.a. 17 MYA) in the central Balkans, and for which cryptic lineages are locally endemic due to paleo-habitat fragmentation and a complex phylogeographic history [36,38]. Furthermore, the recent biogeographical study of the pan-European morphospecies *Gammarus pulex* [41–43] and *Gammarus fossarum* [44] show high levels of cryptic lineages diversity, with diversification starting some >10 MYA. The crown age of the genus *Gammarus* itself has been estimated at 65 MYA [41]. All this offers the opportunity to extend the analysis of *Nosema* diversity to a wider set of European gammarid species and to decipher the age of infection in this group of hosts.

Besides the aforementioned advantages, the study of the phylogenetic diversity of microsporidian parasites faces some problems with the resolution of available genetic markers. The small subunit ribosomal DNA marker (SSU) is routinely used to PCR-screen hosts for microsporidian infections. SSU sequencing data proved to be very useful for phylogenetic reconstructions at higher taxonomic levels and for distinguishing major microsporidian clades (*e*.*g*. genera) [45], and sometimes also for resolving phylogenetic relationships at lower taxonomic levels [40,46]. However, the use of SSU showed some unresolved phylogenetic relationships [e.g. *Dictyocoela duebenum* / *muelleri* [46], and generated ambiguous results for the *Nosema* genus. On the one hand, it enabled formal redefinition of the two genera *Nosema* and *Vairimorpha* within the family Nosematidae [5]. On the other hand, phylogeography of *N*. *granulosis* in *G*. *roeselii* based on SSU showed a low level of genetic variation despite a pan-European sampling [31]. Therefore, a more variable marker is needed to unravel the phylogeography of *N*. *granulosis* and the evolution of *Nosema*-amphipod associations. The genomes of the few economically relevant insect-infecting *Nosema* spp. have been sequenced [47–49], as well as, recently, the genome of *N*. *granulosis* [50]. Based on these available genome data, the large subunit of the RNA polymerase II (RPB1) gene has been shown to be a suitable marker to unravel microsporidian phylogenetic relationships in cases where SSU was of limited value [5,51–53]. RPB1 is used for all mRNA synthesis in eukaryotes [54], displaying a high level of synonymous variation. Its higher level of genetic variation compared to SSU was already emphasized in a previous study on Microsporidia [51].

The aim of the present study was to deepen our understanding of the evolutionary history of *Nosema* lineages infecting amphipod crustaceans, focusing mainly on *Gammarus* species, by using both SSU and RPB1 markers. Specifically, we first aimed at elucidating the extent of molecular variation associated with *Nosema* infections in amphipods (i.e., could all lineages identified as *Nosema* based on SSU be considered as *Nosema granulosis sensu stricto*, or are they divergent enough to be considered as different species?). Second, we wanted to infer host specificity and explore evolutionary history scenarios that may explain the diversity of gammarid-infecting *Nosema*. Finally, as new divergent lineages of *Nosema* were detected over the entire distribution area of a new host, *Gammarus balcanicus*, the possible existence of sex ratio distortion associated with vertical transmission was tested, using the crude proxy of an infection bias in females (as already used in [20]). Such a test allowed us to explore whether sex ratio distortion is a trait that appeared more than once in the evolution of amphipod *Nosema*.

## Materials and methods

### Overview of dataset composition for *Nosema* infecting amphipods

Our dataset combined information on 316 *Nosema*-infected amphipod individuals gathered from three sources. The first source was data from the literature for which both SSU and RPB1 sequences were available. The second source was data for which SSU information is available from the literature or GenBank and for which RPB1 information was tentatively gained from the original DNA samples as part of this study. The third source was *de novo* sequencing data of parasite SSU and RPB1 from individuals detected as infected by *Nosema* by SSU PCR screenings (S1 and S2 Tables).

The first source is very limited, comprising of only two pairs of sequences, one associated with infection of *G*. *duebeni* [55] and one associated with *G*. *roeselii* [50]. The second source included samples of *G*. *fossarum*, *Niphargus schellenbergi* from Luxembourg and *Niphargellus arndti* from Poland [34,56], *G*. *pulex* from Poland (Wroblewski unpublished), *G*. *pulex* and *G*. *fossarum* from Germany [57], *G*. *duebeni* from Ireland [58] and *G*. *roeselii* from France [23]. A large number of amphipod individuals from different countries were available for three taxa: *Dikerogammarus villosus* [59], *G*. *roeselii* [31] and *G*. *balcanicus* [60] from 34, 94 and 88 sites, located in 8, 19 and 13 countries, with 1436, 1904 and 2225 host individuals tested, respectively. The third source was associated with three host taxa we sampled during three local-scale surveys: *G*. *duebeni* (sites KER and ROS in Brittany, France), *G*. *balcanicus* (site SK in Slovakia), and *G*. *pulex* (5 sites in France, one in Germany and one in Poland). In addition, host individuals from two large-scale surveys we conducted on *G*. *fossarum* and *G*. *balcanicus* were included (S1 Table). In *G*. *balcanicus* (unlike other species), the sex of individuals was noted during dissection (S1 Table). This allowed us to test if females are more often infected than males, which is an indication of sex-biased infection by the parasites and suggestive of VT mode and sex ratio distortion, as previously shown in *G*. *duebeni* and *G*. *roeselii* [20,23].

### *Nosema* infection status based on SSU ribosomal DNA sequencing

All host individuals without previous information on microsporidian infection were first screened to assess possible infection with *N*. *granulosis* based on the SSU marker. SSU was used for both molecular screening for microsporidian infection based on specific PCR primers for Microsporidia and assignment to *Nosema* based on BlastN searches [61] of sequenced PCR products against the sequences available in GenBank. All molecular lab work conditions were as described in [31].

### Amplification and sequencing of *Nosema* RPB1

All *Nosema*-infected individuals (based on SSU) were selected for RPB1 amplification (276 individuals). As all published primers tested [51,52,62,63] failed to produce reliable PCR products of our samples, we designed new primers. Five RPB1 sequences were manually aligned, including three *N*. *granulosis* sequences (associated with infection of *G*. *duebeni*: DQ996233 and EF119339 [51], and *G*. *roeselii*: SBJO01000442 [50]), and two sequences from *Nosema* infecting insects: *N*. *bombycis* (DQ996231 [51]) and *Nosema empoascae* (DQ996232 [51]). Variable regions of interest for phylogenetic reconstruction and conserved regions suitable for primer design were identified from the alignment. Two sets of degenerated primers were designed using Geneious 10.2.2 [64], targeting two non-overlapping fragments, named F2 and F4, starting at positions 386 and 1161 on the RPB1 gene of the genome SBJO01000442, respectively (S1 Fig). The F2 primers were 5’-GKT GTG GRA ATA AAC AGC-3’ (forward, F2f) and 5’-TCT ACT CTC TTM CCC ATA AG-3’ (reverse, F2r), generating a 520 bp-long amplicon. The F4 primers were 5’-GAA AGA CAC ATG CAG RAT G-3’ (forward, F4f) and 5’-TTC CWG ACA TGA TYT CTC C-3’ (reverse, F4r), generating a 640 bp-long amplicon.

PCRs were performed in a volume of 30 μl, containing 2.5 mM MgCl_2_, 0.5 units of 5 PRIME HotMaster Taq DNA polymerase (Qiagen, Hilden, Germany), 0.2 μM dNTPs (MP Biomedicals Europe, Illkirch, France), 0.2 μM of forward and reverse primers each (Eurofins Genomics, Ebersberg, Germany) and 2 ng DNA template. Amplification conditions were as follows: an initial denaturing phase at 94°C for 2 min, 35 cycles at 94°C for 20s, annealing temperature was 50°C for each fragment for 20 s and extension was at 65°C for 30 s, with a final extension step at 65°C for 5 min. PCR products were purified and sequenced directly with the BigDye technology (Genewiz, Leipzig, Germany) using the forward PCR primers. The chromatograms showed no ambiguity in sequences and all the detected double peaks were of high quality. Nevertheless, seven PCR products were bi-directionally sequenced to test for uncertain nucleotide position associated with double peaks in chromatograms, and 17 products (from populations AL56, BF05, GR11, H05, NIT, PA and PL8 (S1 Table), were sequenced twice with the forward primer to confirm double peaks.

Using Geneious 10.2.2 [64], raw sequences were checked for microsporidian RPB1 specificity via BlastN search [61] against sequences available in GenBank. Each sequence was manually edited, and double peaks were called following the International Union of Pure and Applied Chemistry (IUPAC) degenerate nucleotide code. The RPB1 gene is known to be a nuclear single-copy gene in *Vairimorpha necatrix*, a close relative of *Nosema* [62], thus excluding paralogy as an explanation for the observed double peaks. Instead, they could result from double infections with *Nosema* individuals harbouring different haplogroups or from single infections by heterozygous individuals. The F2 and F4 sequences were trimmed to the final size ranges of 282 to 455 bp and 280 to 562 bp, respectively, based on the quality level of the sequences. The RPB1 sequences were translated into amino acids to confirm the absence of stop codons (S1A Fig). Sequences were aligned after concatenation using MAFFT7.388 software [65,66] with the E-IONS-I algorithm using the legacy gap penalty option, incorporated in Geneious 10.2.2 [64]. To eliminate introns, translated amino acid versions were also used as a backbone based on the reference the RPB1 sequence AF060234 from *Vairimorpha necatrix*.

### Haplogroup definition and phylogeny reconstruction for microsporidians

As SSU and RPB1 sequence length was heterogeneous among individuals, definition of haplotypes *sensu stricto* was not possible. Following the approach described in [31], sequences of each marker were assigned to haplogroups as follows: two sequences were clustered in one haplogroup based on 100% pairwise nucleotide identity in their shared part, each haplogroup being defined by diagnostic SNPs [31]. Only few sequences could not be assigned to haplogroups, due to reduced sequence length and lack of diagnostic features (S2 Table). Some RPB1 haplogroups showed three short deletions in the F4 fragment (S1B Fig). Finally, the F2 or F4 fragments were missing for some individuals, because amplification failed, and were coded as missing data (“N”).

The longest sequences of each haplogroup, were used for Bayesian phylogeny reconstruction (S2 Table). Haplogroup alignments used for building trees are provided in the S1 Appendix. Compared to SSU, nucleotide variation for the RPB1 marker was high, although mostly synonymous. Consequently, all codon positions were used for phylogenetic analyses.

For phylogenetic analyses, sequences of *Nosema* spp. infecting amphipods were complemented by a set of 11 species of *Nosema/Vairimorpha* spp. infecting insects, according to the taxonomic revision of [5], for which both SSU and RPB1 markers are available. We further included the two following *Nosema* species (both infecting crayfish) in our analysis: *Nosema cheracis*, infecting the Australian crayfish species *Cherax destructor* introduced in Europe and *Nosema austropotamobii*, infecting *Austropotamobius pallipes*, a western European freshwater crayfish. *Ordospora colligata* served as the outgroup [67]. All details are given in S2 Table.

Phylogenetic reconstructions were performed separately for SSU and RPB1 using Bayesian inference implemented in MrBayes [68] integrated in Geneious 10.2.2. The best-fitting model of nucleotide substitution was determined with JModelTest-2.1.10. [69]. For both SSU and RPB1 we used the General Time Reversible (GTR) model with gamma-distributed rate heterogeneity (+G) and a proportion of invariable sites (+I). For each marker, four heated chains, each 1,100,000 iterations long, sampled every 100 iterations, were run. Runs reached satisfactory effective sampling sizes (ESS > 200). Fifty percent majority-rule consensus trees were constructed after removal of 10% ‘burn-in’ trees. For the RPB1 phylogeny reconstruction, a maximum likelihood-tree was also constructed in MEGA version 11 [70] using the GTR+G+I model and node support was assessed with 1,000 bootstrap replicates.

### Molecular species delimitation

To explore the number of molecular operational taxonomic units (MOTUs) that may represent potential species within the *Nosema* clade, we applied two methods: Assemble Species by Automatic Partitioning (ASAP) [71] and the Poisson tree processes (PTP) model [72]. ASAP was conducted using the ASAP webserver (https://bioinfo.mnhn.fr/abi/public/asap/ accessed on June 2, 2023) based on the distance matrix generated through IQ-TREE analysis [71]. ASAP divides species partitions based on pairwise genetic distances, and that was why the dataset for this analysis included only the sequences covering both fragments of RPB1 (F2 and F4). ASAP also computes a probability of panmixia (p-val), a relative gap width metric (W), and ranks results by the ASAP score: the lower the score, the better the partitioning [71].

**Fig 1:**
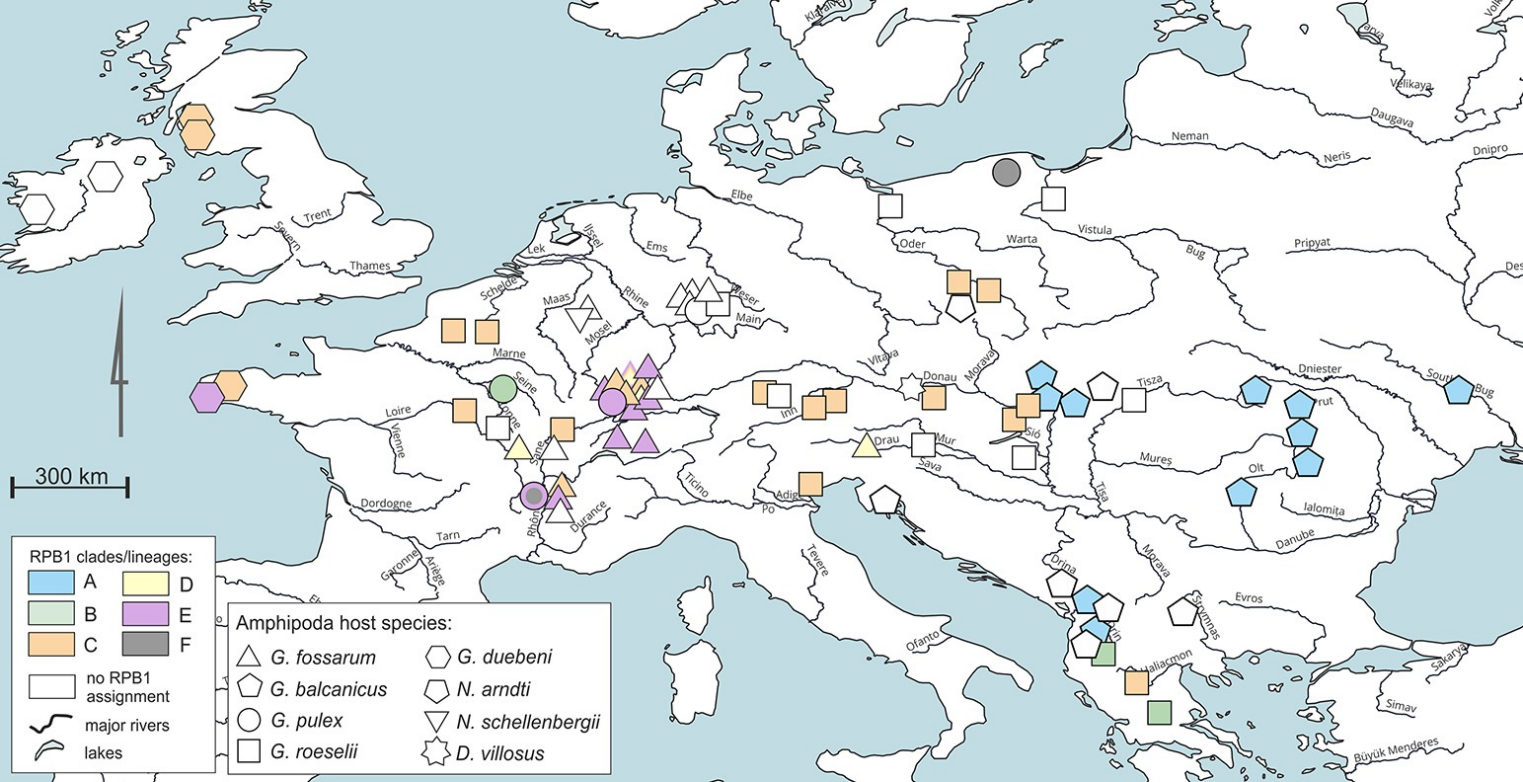
Geographic distribution of *Nosema* spp. infecting a range of amphipod hosts based on data listed in S1 and S2 Tables. Assignment to clades or lineages is based on RPB1 (see Fig 3). The infection of *Nosema* sp. in *Eulimnogammarus verrucosus* from the Lake Baikal, Russia is not shown. The sites were plotted on a map from Natural Earth resources in QGIS 3.32.0-Lima [73].

PTP modelling was performed using the PTP web server (https://species.h-its.org/; accessed on June 1, 2023) with the Bayesian implementation (bPTP), which adds Bayesian support (pp) values for putative species to branches in the input tree. The PTP method infers speciation events based on a shift in the number of substitutions between internal nodes [72]. Analysis was run with 500 000 iterations, the run was examined and showed convergence. The sites reported on Figs 1 and S2 were plotted on a map from Natural Earth resources in QGIS 3.32.0-Lima [73].

### Testing sex-biased infection pattern

In *G*. *balcanicus*, 334 males and 310 females from 16 populations were tested for estimating the prevalence of *Nosema* (after removing the two populations in which no females were tested, S1 Table). A General Linearized Model (binomial distribution using a Logit transition function) was executed, analyzing the effect of sex and population as factors, and their interactions, on the infection rate by *Nosema*. A bias of infection towards females should be associated with sex ratio distortion [20,23].

## Results

Based on SSU sequences, 316 individuals from nine amphipod species were found to be infected by *Nosema* spp. (S1 and S2 Tables). Our new census yielded 181 new *Nosema* sequences: 89 from *G*. *balcanicus*, 39 from *G*. *duebeni*, 47 from *G*. *fossarum*, five from *G*. *pulex* (S1 Table). They extend a set of 136 sequences previously determined as *Nosema* (99 from *G*. *roeselii*, 18 from *G*. *pulex*, 7 from *G*. *duebeni*, 6 from *G*. *fossarum*, two from *D*. *villosus*, two from *Eulimnogammarus verrucosus*, one from *N*. *arndti* and one from *N*. *schellenbergi*) (S1 and S2 Tables).

**Fig 2:**
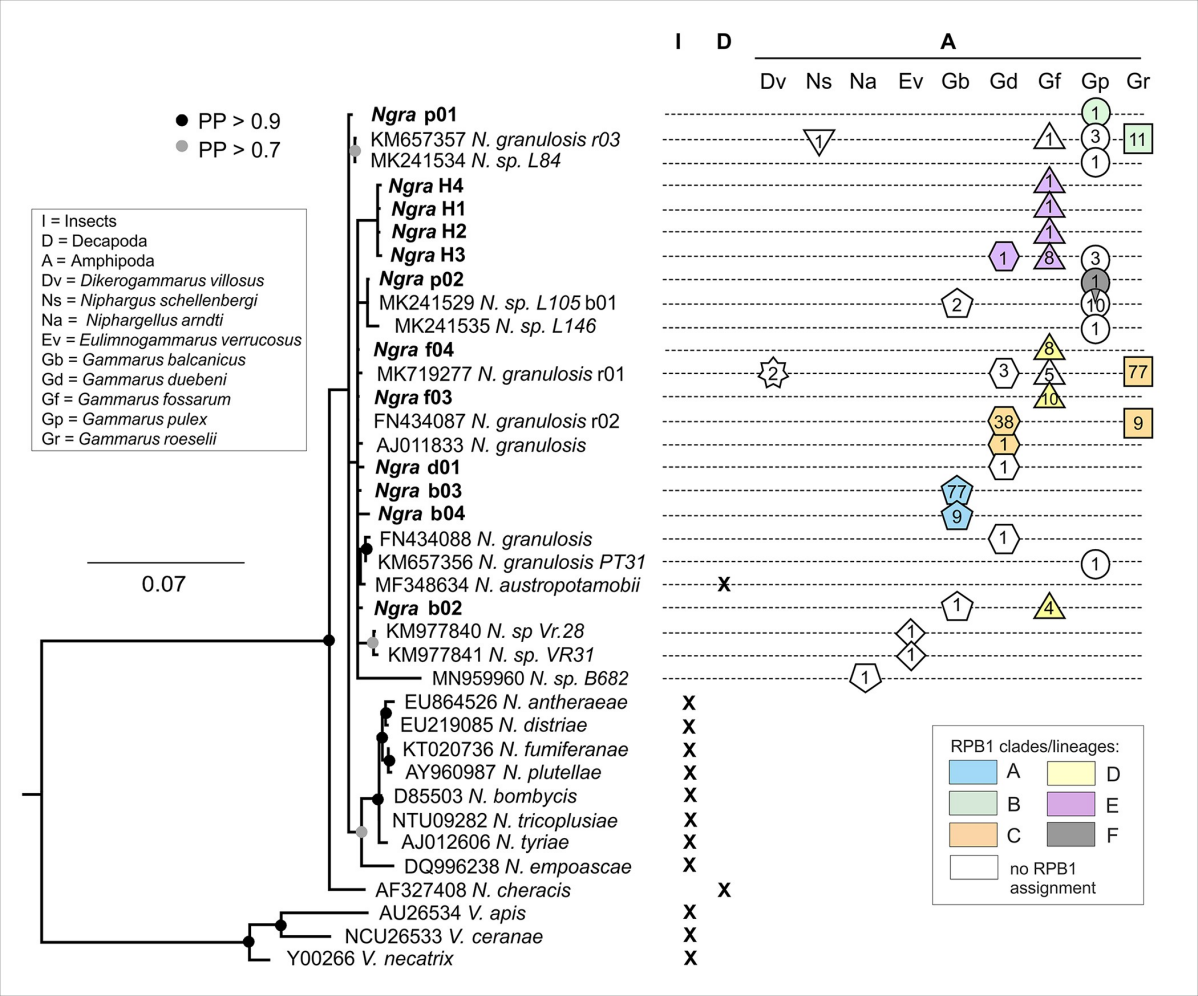
Bayesian phylogenetic reconstruction of *Nosema* spp. based on small ribosomal subunit (SSU) rDNA, and their association to different host species. *Ordospora colligata* (AF394529) was used as an outgroup (not shown). Labels include: Genbank accession number (for previously published sequences), microsporidian species name as given in [5] and for the newly produced sequences the haplogroup name is given as in S1 and S2 Tables. An X denotes an association and a figure denotes the number of individuals infected, each amphipod species being represented by a given geometric symbol (see also Fig 1). Colour code refers to lineages identified by the RPB1 marker (see Fig 3). PP: Bayesian Posterior Probability.

The phylogenetic reconstruction inferred from the SSU marker was to a large extent in line with previously published phylogenetic reconstructions (e.g. [31]), except that the clade containing the *Nosema* spp. found in amphipods also included a crayfish-infecting species (Fig 2). Most haplogroups (18/24) were found specifically infecting a single host species, while six were found in two to four host species.

Amplification success of the F2 and F4 fragments of RPB1 gene was variable, including no success (113 ind., 41%), single fragment amplified (57 ind., 21%) or both fragments amplified (105 ind., 38%). Therefore, we newly obtained RPB1 sequence information for 163 individuals (depending on DNA availability and sequencing success), including 60 from *G*. *roeselii*, 45 from *G*. *balcanicus*, 38 from *G*. *fossarum*, 15 from *G*. *duebeni* and 4 from *G*. *pulex*. To this set, two RPB1 sequences of VT, feminizing, *N*. *granulosis* infecting *G*. *duebeni* from Scotland and Whales [10,55] were added which matched the F2 and F4 regions of RPB1 (GenBank accession numbers, DQ996233 and JX213747). The geographic distribution of the parasites for which the RPB1 sequences were obtained is shown in Fig 1.

**Fig 3:**
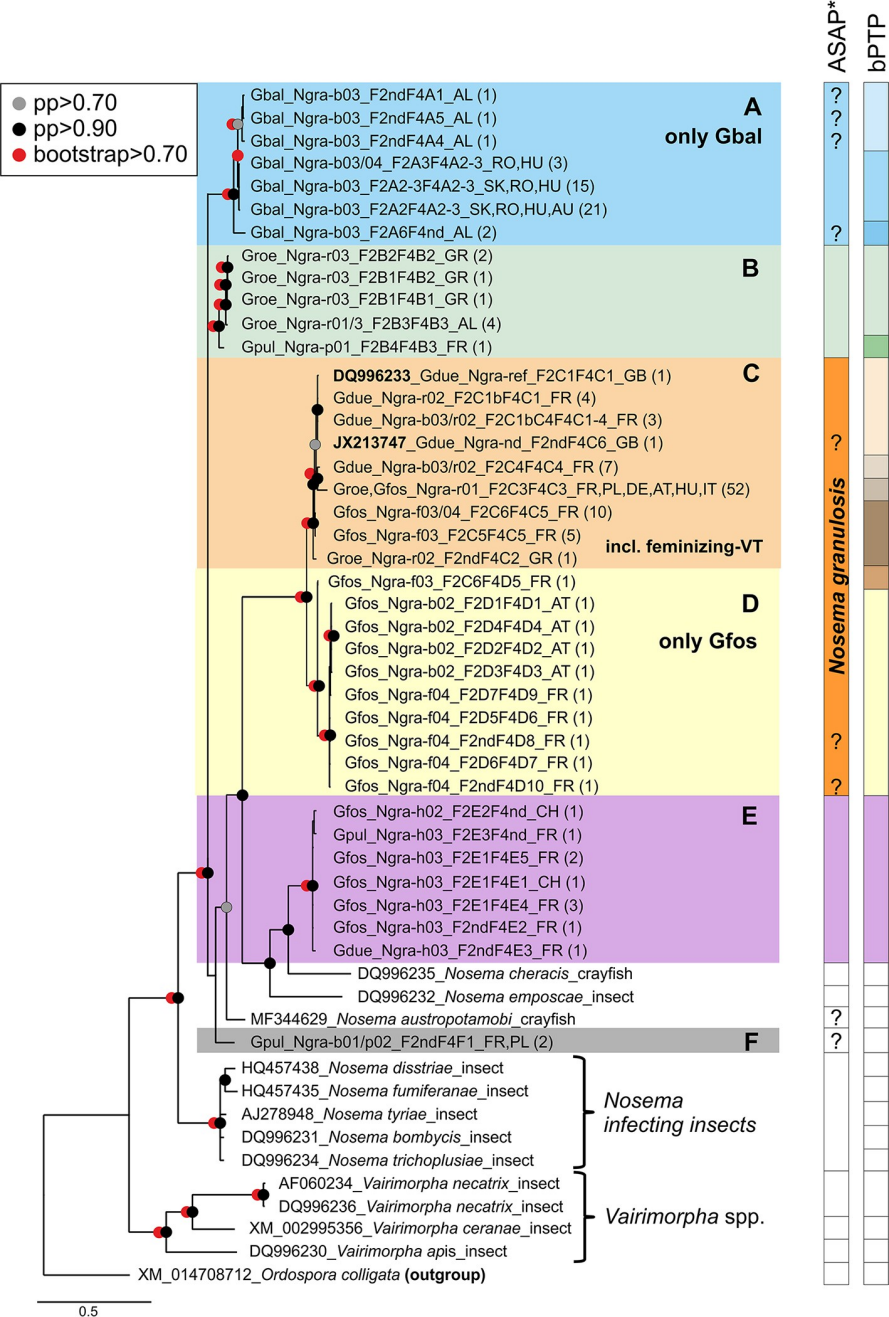
Bayesian phylogenetic reconstruction of *Nosema* spp. based on fragments F2 and F4 of the RPB1 gene. For parasite sequences found in amphipods labels include: the GenBank accession number in case of published sequences, the host species name (abbreviated for gammarids, see below), the abbreviated SSU haplogroup name (as in S2 Table and Fig 2), the RPB1 haplogroup name, population name and the number of host individuals infected. Other sequences from Genbank are representatives of further *Nosema* and *Vairimorpha* species (see S2 Table). PP = Bayesian Posterior Probability, nodes supported by the Maximum Likelihood tree by bootstrap values higher than 0.7 are indicated by red dots. Bars annotated on the right represent results of the molecular species delimitation ASAP and bPTP methods. *– ASAP analysis was performed using only the sequences covering both F2 and F4 RPB1 fragments; ? –haplogroup excluded from the ASAP analysis because of lack of F2 or F4 fragment. Abbreviations for gammarid species names: Gbal–*Gammarus balcanicus*, Gdue–*Gammarus duebeni*, Gfos–*Gammarus fossarum*, Gpul–*Gammarus pulex*, Groe–*Gammarus roeselii*.

Alignment of RPB1 sequences from *Nosema* infecting amphipods revealed 39 haplogroups (Fig 3) showing a higher variability compared to the phylogenetic relationships among haplogroups based on SSU sequences (Fig 3). The amphipod-infecting *Nosema* spp. were all within a well-defined and supported clade, which was the sister to a clade of insect-infecting *Nosema* spp. (*sensu* [5]). Based on the available sequences, the amphipod-*Nosema* clade also contained three sequences from non-amphipod hosts (Fig 3). All but one sequence of amphipod-infecting *Nosema* spp. could be assigned to five well-supported clades (named A to E, Fig 3). The single remaining sequence is associated with a branch (termed F) of uncertain phylogenetic position (highlighted in grey in Fig 3).

Parasites from clade A were found only in *G*. *balcanicus* (Fig 3), despite extensive sampling. These *Nosema* strains, particularly F2A2-3F4A2-3 and F2A2F4A2-3, were found in numerous populations, exhibiting high prevalence in some of the populations (see S1 Table), and their geographic distribution covered the entire geographic range of their host (Fig 1). However, they were not found in other *Gammarus* species from the close area, and were particularly absent in *G*. *roeselii*, the other abundant host species overlapping *G*. *balcanicus* distribution (Fig 1). We found no clear pattern for co-diversification between *G*. *balcanicus* and these *Nosema* parasites. We were not able to run a formal co-diversification analysis, because the asymmetry of amplification of F2 and F4 fragments among some hosts (S2 Fig) prevented the construction of a complete matrix of genetic distance among parasite haplogroups of this relatively small clade. We nevertheless observed only little congruence of phylogenies between hosts and parasites (S2 Fig). We tested whether, with our dataset, we could provide clues suggesting the presence of VT/sex ratio distortion in *Nosema* clade A infecting *G*. *balcanicus*. A General Linearized Model analysis showed that the interaction sex*population was non-significant and the corrected Akaike Information Criterion (AICc) of the whole model was quite high (LR χ2 = 18.03, 15 df., P = 0.26, AICc = 553.19), so the interaction was removed from the model. The non-significance of this interaction means that globally there was no difference in the infection rate between sexes among the different populations. The model including sex and population was globally significant (LR χ2 = 124.59, 16 df., P < 0.0001, AICc = 538.60). The infection rate significantly differed among populations, ranging from 4.2% (in populations AL57 and AL69) to 64.6% (in population UA03) (S1 Table). In total, males were more infected than females (26.9% vs. 14.8%, respectively, LR χ 2 = 17.67, 1 df., P < 0.0001). In most populations, there was no significant difference between sexes (12 populations), but in four populations, males were significantly more infected than females (populations AL56, H07, H09, H11).

Parasites from clade B were found in *G*. *pulex* and *G*. *roeselii* hosts. *Gammarus roeselii* parasites were restricted to south-eastern Europe (Greece and Albania), while the single haplogroup from *G*. *pulex* was recorded in France (Fig 1). Clades A and B were more distantly related to clades C, D and E.

Clade C included the parasites infecting *G*. *duebeni*, i.e. the host in which *Nosema granulosis* was first described as a vertically-transmitted, feminizing sex ratio distorter [10]. Interestingly, clade C also included *Nosema* infecting *G*. *roeselii*, in which vertical transmission and feminization has also been demonstrated [23,24]. Overall, clade C haplogroups were widespread all over Europe, and it must be noticed that a single haplogroup (F2C3F4C3) was frequent (52 occurrence) and detected in various populations of *G*. *roeselii* from six countries and in one French population of *G*. *fossarum* (Figs 2 and 3, S2 Table). Clade D, which appears to be closely related to clade C, consisted of highly similar haplogroups of *Nosema* that were solely found in *G*. *fossarum* (Fig 3, S3 Table). Clade D was restricted to France and Austria, and these parasites infected nine individuals from four populations. All haplogroups from clades C and D shared two short deletions in the F4 fragment (deletions of 27 and 15 bp) (S1 Fig) and they were not distinguishable using the SSU marker (Fig 2). Haplogroups of clade C included a supplementary 15 bp-long deletion.

Finally, clade E consisted of parasites infecting three host species (*G*. *fossarum*, *G*. *pulex* and *G*. *duebeni*) sampled at French and Swiss sites (Fig 1).

In summary, our sequences grouped within clades A, B, C, D, E, with C and D being sister groups, and a single sequence identified as lineage F. The ASAP molecular delimitation analysis revealed four putative species within *Nosema* infecting amphipod crustaceans (excluding lineage F), mostly overlapping with the aforementioned clades, but C and D grouped as one species, here interpreted as *Nosema granulosis sensu stricto* (Fig 3). On the other hand, bPTP overall suggested from 19 to 29 putative species, with partitioning to 25 species with the highest support (lowest posterior probability 0.50) (S2 Appendix), and 13 species within *Nosema* found in amphipods (Fig 3).

## Discussion

Our results indicate that various microsporidian lineages of the genus *Nosema*, not only *N*. *granulosis*, are abundant, widespread and diverse in European gammarid amphipods. Thus, we deepen our knowledge of host range for this parasitic genus beyond the already well-known insects and terrestrial hosts (e.g. [74–76]). Indeed, our phylogenetic reconstructions unambiguously grouped freshwater amphipod parasites as a sister group to Lepidoptera and Hymenoptera-infecting *Nosema* (*sensu* [5]). However, we cannot formally conclude that amphipod *Nosema* are monophyletic. The phylogenetic tree reconstruction based on the RPB1 marker suggests that *Nosema* spp. found in freshwater crustaceans are not limited to amphipod hosts and include one terrestrial species, *Nosema empoascae*. Furthermore, *Nosema* isolates infecting crayfish are nested within those infecting amphipods. This could suggest either that there has been an ancient *Nosema* infection in Malacostraca (the crustacean group to which amphipods and decapods belong), or a freshwater origin of this *Nosema* clade, with horizontal transfers between host species. The divergence among crustacean *Nosema* strains reported here could appear low compared to host divergence (Malacostraca diversified around 300 MYA [77]). Crustaceans colonized freshwater at diverse times independently [78]. If so, the most likely explanation would be horizontal transfer of parasites. However, the molecular phylogenetic reconstructions of the hosts and the parasites could not be compared directly since different genetic markers were used. For example, the New-Zealand amphipod hosts and their microsporidian parasites (*Dictyocoela*) seemed to have initiated their diversification simultaneously several MYA, but their genetic divergence appears to have reached different levels [35]. The presence of the insect-infecting *N*. *empoascae* within the *Nosema* lineage infecting crustaceans is more puzzling and may reflect horizontal transfer between crustaceans and insects. Consistently, horizontal transfer of *Wolbachia* intracellular microorganisms between insects and crustaceans has previously been suggested [79]. However, owing to the strict terrestrial life cycle of the insect host of *N*. *empoascae* (*Empoasca fabae*, a small leafhopper of the Cicadellidae family [80]) a recent host shift is ecologically difficult to conceive in the present state of knowledge. In addition, this parasite seems to be vertically transmitted in its host [81], which may further reduce the likelihood of horizontal transfer between hosts. However, the existence of additional vector/intermediate hosts such as water insects or terrestrial insects drowning in water cannot be excluded, as suggested for *Nosema cheracis* [11], even if no data is available up to now. Besides, microsporidian samples presently available for insect hosts are far from representative of the global diversity of *Nosema* infecting these hosts. Thus, future studies are necessary to address the problem of the potential transfer of *Nosema* between aquatic and terrestrial ecosystems.

The improved phylogenetic resolution of the RPB1 marker compared to SSU allowed us to distinguish five *Nosema* clades infecting amphipods [31,50]. Clade A was found only associated with *G*. *balcanicus*, whose biogeographical history is well documented for both host and other microsporidian parasites [36,39,40]. In this case, host specificity may be proposed. Indeed, clade A parasites infected individuals in several locations throughout the entire host range. In addition, they colonized diverse host cryptic lineages (S2 Fig.) but they were not found in other *Gammarus* spp., conversely to most other *Nosema* parasites revealed in this study. An infection by *Nosema* clade A therefore possibly occurred anciently in *G*. *balcanicus* and subsequently spread further during host radiation. However, unlike other microsporidian parasites [40], no clear co-diversification pattern was found between *Nosema* clade A and *G*. *balcanicus* (S2 Fig). Therefore, ancient infection followed by several horizontal transfers may explain the present distribution of *Nosema* in *G*. *balcanicus*. Parasites belonging to clade D were restricted to a few lineages of the *G*. *fossarum* species complex [44]. However, the range of the host *G*. *fossarum* is pan-European, and since we did not have access to the whole geographic range for our survey, we cannot firmly conclude that the parasites of clade D are specific to this host. Because of the low diversity within this clade, it is likely that these infections were recently acquired by the host species.

Prior to this study, microsporidians from gammarids showing high score values by BlastNN with *Nosema granulosis* isolated from *G*. *duebeni* based on SSU sequences were generally assigned either to *N*. *granulosis* [31,34], to *Nosema* sp. or to *Vairimorpha* sp. [82]. Here we found that the vertically-transmitted (VT) and feminizing *N*. *granulosis* described from *G*. *duebeni* [10,19–21] correspond to clade C, also includes the closely related *Nosema* from *G*. *roeselii*, which likely is a VT sex ratio distorting parasite [23,24]. These infections are characterized by a high number of infected individuals in each host species, numerous infected populations, and few haplogroups in each species (e.g., in *G*. *roeselii*, 77 individuals from 12 populations share the same RPB1 variant). As noted in [31], all these traits are compatible with the dynamics of VT-feminizing microsporidians. Their spread in host populations is enhanced by parasite-induced female-biased sex ratios of host progenies, which provides a selective advantage for infected vs. uninfected females [30]. Theoretical models suggest that female-biased sex ratios result in populations with high numbers of individuals and high demographic dynamics that may outcompete non-infected populations [30]. Thus, the presence of this VT *N*. *granulosis* infection might have facilitated the recent spread of the host species, *G*. *roeselii*, in western Europe [32,37], by directly enhancing host invasion success through increased rates of population growth, as suggested for another amphipod *Crangonyx pseudogracilis* and its VT-parasite, *Fibrillanosema crangonycis* [83,84]. Because all *Nosema* belonging to clade C are genetically very similar to the originally described *N*. *granulosis* (0.02 ± 0.003 average genetic divergence over all RPB1 sequence pairs), we propose that all parasites clustering into clade C should be referred to as *N*. *granulosis*. Furthermore, because i) clades C and D are in a close sister group relationship, ii) clade C contains sequences linked to type material used for *N*. *granulosis* description [10], iii) the monophyly of clades C +D is statistically supported, and iv) clade C + D is also supported by the ASAP analysis, we propose that clades C and D should be seen as *Nosema granulosis sensu stricto*. By contrast, we suggest that parasites grouped into clades A, B and E, and lineage F may be candidates to the status of new *Nosema* species, based on their genetic divergence supported by the species delimitation analysis. Remarkably, this divergence is larger than the range of divergence observed for *Nosema* taxa from insects. It may be explained by the differences in mutation rate and evolutionary dynamics between *Nosema* spp. infecting gammarids and insects. In our interpretation of the number of putative *Nosema* species infecting gammarids, we followed a more conservative approach: we based our outcomes on the ASAP results assuming fewer putative species than the bPTP analysis. There are many studies demonstrating that ASAP is often in congruence with morphological/biological species delimitation while bPTP method tends to overestimate the species number [e.g. 46,85,86]. However, more studies including ultrastructural analysis are needed to formally describe those clades as putative species. We also cannot exclude that our new lineages can be *Nosema* spp. already described from different amphipods, including *G*. *pulex*, based solely on morphology (light microscopy for older observations) [13–17,87]. However, morphological descriptions of *Nosema* can be misleading [5], and using an integrative approach is advisable and ultimately could lead to taxonomic revisions [88].

Interestingly, most haplogroups involving *Nosema* from clades B, D and E are present in a single or few individuals within the same population. Because of the sharp contrast of this infection pattern compared to clade C, it does not seem reasonable to us to propose that parasites of clades B, D, and E are feminizing and VT microsporidia. Contrastingly, infections in *G*. *balcanicus* hosts belonging to clade A show an infection pattern similar to those of clade C. For example, sequences in populations from Slovakia, Hungary and Romania show closely-related or similar RPB1 haplogroups infecting numerous individuals (> 70) in a dozen of populations all over the host geographic range. This pattern therefore resembles the VT-feminizing *N*. *granulosis* infection patterns in *G*. *roeselii* (Figs 2 and 3). However, the analysis of infection patterns relative to sexes showed that there were no significant differences in prevalence between sexes in most populations. Yet, parasites that have been shown to be sex ratio distorters—such as *N*. *granulosis* in *G*. *duebeni* and *G*. *roeselii* or *Wolbachia pipientis* in the isopod *Armadillidium vulgare*—are always more frequent in females than in males (which makes sense given that the parasites convert infected males into females) [20,23,89]. The high prevalence and above all the homogeneity of *Nosema* haplogroups across the geographic range of *G*. *balcanicus* is therefore puzzling. Based on this survey and previous evidence, VT and feminization in *Nosema* infecting amphipods therefore currently appear to be limited to *N*. *granulosis* belonging to clade C. It is noteworthy that clade C also contains parasites infecting *G*. *fossarum* individuals, which opens the possibility that VT-feminization might be found in a larger spectrum of amphipod species than previously thought.

In conclusion, our study illustrates the interest of using the RPB1 marker for the study of *Nosema* parasites, allowing elucidating the parasitic richness in their gammarid hosts. We found that *Nosema* diversity is much higher in amphipods than previously thought, and that the association appears to be ancient in this host group. Furthermore, the new host species found to be infected by *N*. *granulosis sensu stricto* opens the possibility that VT-feminization may be present in a larger spectrum of amphipod species. Finally, given that gammarids are keystone species in the functioning of freshwater environments, with their functions being affected by microsporidian infections [90–93], it is of crucial importance to correctly identify host-parasite relationships by means of reliable markers.

## Supporting information

S1 Fig A. Overview of the amino-acid alignment for the RNA polymerase II largest subunit (RPB1) gene haplogroups identified in amphipods. Grey colour stands for absence of amino acids. B. Overview of the nucleotide alignment for the RPB1 gene haplogroups, for two fragments (F2 and F4). Grey colour stands for either absence of PCR product or shorter sequences for a given fragment.
(PDF)

S2 Fig **A.** Details of Clade A of *Nosema* phylogenetic reconstruction. Names in black are *Nosema* haplogroups, names in red or blue are names of host clades to which they are associated. Legends are similar to Fig 3 in the paper. Host clade names are given from the *Gammarus balcanicus* phylogenetic tree simplified from [36] presented in (**B**). N and S represent the two major *G*. *balcanicus* groups that differentiated around 18 MYA. **C**. Map showing sites where host and parasites are coming from, where the limit between N and S host groups were redrawn after [36]. Lines between A and B trees indicates the position of host individuals infected by *Nosema* haplogroups on host phylogenetic tree. The sites were plotted on a map from Natural Earth resources in QGIS 3.32.0-Lima [73].
(PDF)

S1 Table A list of 85 sites where *Nosema* spp. were recorded in amphipod crustaceans. The names of the SSU haplogroups are as in Fig 2 and for RPB1 clades/lineages as in Figs 1 and 3.
(XLSX)

S2 Table A list of samples used in the study, with host, site and country name, as well as GenBank Accession numbers given. Sequences in blue are samples of *Nosema* infections outside the amphipod hosts, used for the phylogenetic reconstructions. **sequences too short to be deposited in GenBank (see S2 Appendix). References: Refence list is provided in the spread-sheet REFERENCES. DS: Direct submission to Genbank, no published article associated.
(XLSX)

S3 Table Estimates of Average Evolutionary Divergence over Sequence Pairs within Groups. The number of base substitutions per site from averaging over all sequence pairs within each group are shown. Analyses were conducted using the Kimura 2-parameter model. This analysis involved 36 nucleotide sequences. Codon positions included were 1st+2nd+3rd+noncoding. All ambiguous positions were removed for each sequence pair (pairwise deletion option). There were 1025 positions in the final dataset. Evolutionary analyses were conducted in MEGA11. The presence of n/c in the results denotes cases in which it was not possible to estimate evolutionary distances. SE—standard error. References: Refence list is provided in the spread-sheet REFERENCES.
(XLSX)

S1 Appendix Alignment of F2 and F4 RPB1 fragments of *Nosema* spp., *Vairimorpha* spp. and outgroup used for the Bayesian and Maximum Likelihood phylogeny reconstructions. (TXT)

S2 Appendix Small ribosomal subunit (SSU) rDNA sequences too short (<200bp) to be deposited in GenBank. (TXT)

S3 Appendix Results of the ASAP and bPTP species delimitations. (TXT)
