# Supplementary material for: Widespread infection, diversification and old host associations of Nosema Microsporidia in European freshwater gammarids (Amphipoda)
Source: PLoS Pathog. 2023 Aug 21;19(8):e1011560. doi: 10.1371/journal.ppat.1011560 (PMC10470943; doi:10.1371/journal.ppat.1011560)
Supplement: S1 Fig — A. Overview of the amino-acid alignment for the RNA polymerase II largest subunit (RPB1) gene haplogroups identified in amphipods. Grey colour stands for absence of amino acids. B. Overview of the nucleotide alignment for the RPB1 gene haplogroups, for two fragments (F2 and F4). Grey colour stands for either absence of PCR product or shorter sequences for a given fragment. (PDF) [file ppat.1011560.s001.pdf]

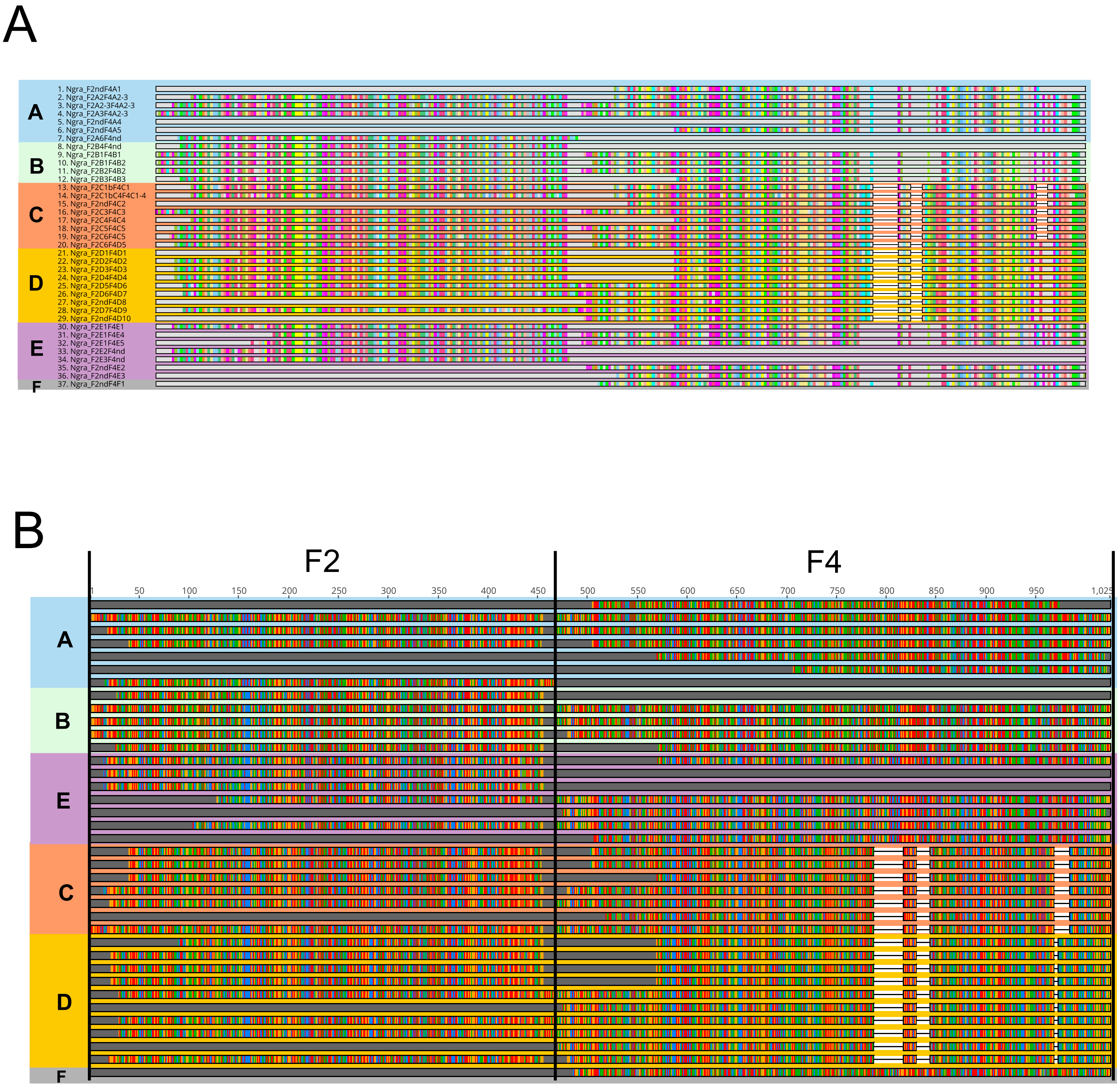

S1 Fig

A. Overview of the amino-acid alignment for the RNA polymerase II largest subunit (RPB1) gene haplogroups identified in amphipods. Grey color stands for absence of amino acides.

B. Overview of the nucleotide alignment for the RPB1 gene haplogroups, for two fragments (F2 and F4). Grey color stands for either absence of PCR product or shorter sequences for a given fragment.
