# Supplementary material for: Widespread infection, diversification and old host associations of Nosema Microsporidia in European freshwater gammarids (Amphipoda)
Source: PLoS Pathog. 2023 Aug 21;19(8):e1011560. doi: 10.1371/journal.ppat.1011560 (PMC10470943; doi:10.1371/journal.ppat.1011560)
Supplement: S2 Fig — A. Details of Clade A of Nosema phylogenetic reconstruction. Names in black are Nosema haplogroups, names in red or blue are names of host clades to which they are associated. Legends are similar to Fig 3 in the paper. Host clade names are given from the Gammarus balcanicus phylogenetic tree simplified from [36] presented in (B). N and S represent the two major G. balcanicus groups that differentiated around 18 MYA. C. Map showing sites where host and parasites are coming from, where the limit between N and S host groups were redrawn after [36]. Lines between A and B trees indicates the position of host individuals infected by Nosema haplogroups on host phylogenetic tree. The sites were plotted on a map from Natural Earth resources in QGIS 3.32.0-Lima [73]. (PDF) [file ppat.1011560.s002.pdf]

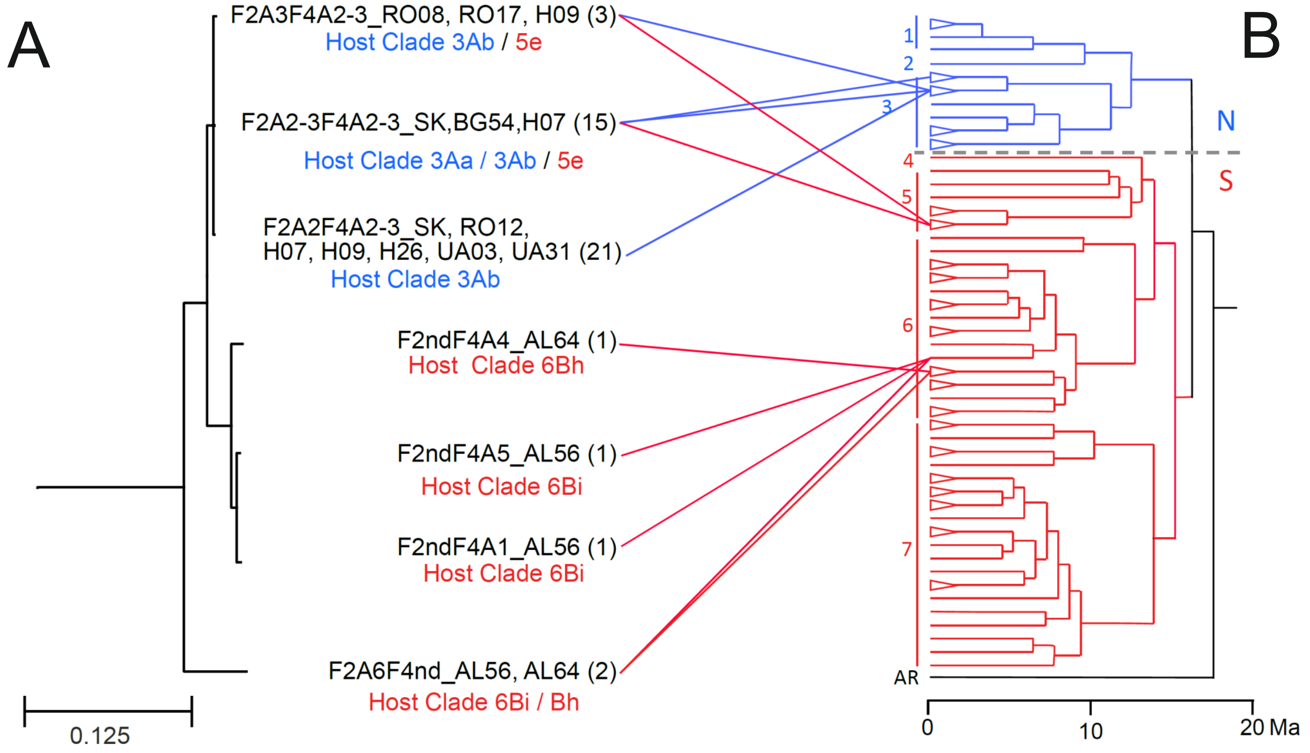

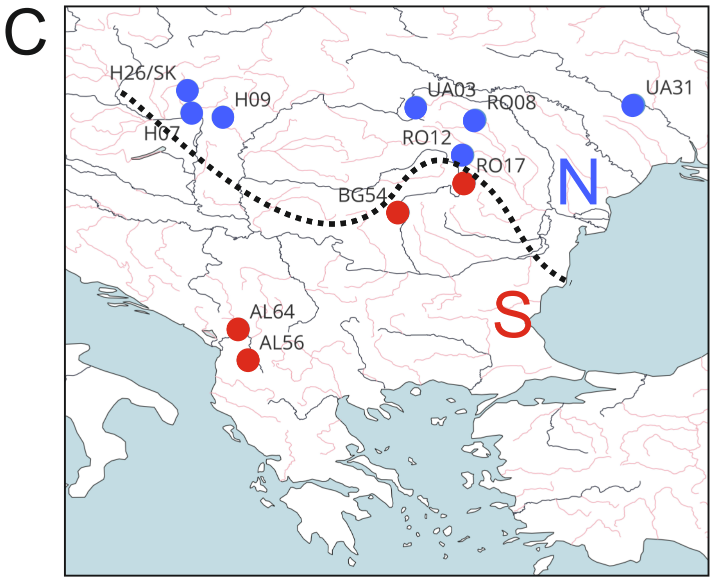

S2 Fig. A. Details of Clade A of *Nosema* phylogenetic reconstruction. Names in black are *Nosema* haplogroups, names in red or blue are names of host clades to which they are associated. Legends are similar to Fig 3 in the paper. Host clade names are given from the *Gammarus balcanicus* phylogenetic tree simplified from [36] presented in (**B**). N and S represent the two major *G. balcanicus* groups that differentiated around 18 MYA. **C**. Map showing sites where host and parasites are coming from, where the limit between N and S host groups were redrawn after [36]. Lines between A and B trees indicates the position of host individuals infected by *Nosema* haplogroups on host phylogenetic tree. The sites were plotted on a map from Natural Earth resources in QGIS 3.32.0-Lima [74].
